# Surgical procedure of intratympanic injection and inner ear pharmacokinetics simulation in domestic pigs

**DOI:** 10.3389/fphar.2024.1348172

**Published:** 2024-01-26

**Authors:** Adele Moatti, Shannon Connard, Novietta De Britto, William A. Dunn, Srishti Rastogi, Mani Rai, Lauren V. Schnabel, Frances S. Ligler, Kendall A. Hutson, Douglas C. Fitzpatrick, Alec Salt, Carlton J. Zdanski, Alon Greenbaum

**Affiliations:** ^1^ Joint Department of Biomedical Engineering, University of North Carolina at Chapel Hill, North Carolina State University, Raleigh, NC, United States; ^2^ Comparative Medicine Institute, North Carolina State University, Raleigh, NC, United States; ^3^ Department of Clinical Sciences, North Carolina State University, Raleigh, NC, United States; ^4^ Department of Otolaryngology-Head and Neck Surgery, University of North Carolina at Chapel Hill, Chapel Hill, NC, United States; ^5^ Department of Biomedical Engineering, Texas A&M University, College Station, TX, United States; ^6^ Tuner Scientific, Jacksonville, IL, United States

**Keywords:** intratympanic, inner ear, fluid simulation, round window membrane, pigs, swine, pharmacokinetics

## Abstract

**Introduction:** One major obstacle in validating drugs for the treatment or prevention of hearing loss is the limited data available on the distribution and concentration of drugs in the human inner ear. Although small animal models offer some insights into inner ear pharmacokinetics, their smaller organ size and different barrier (round window membrane) permeabilities compared to humans can complicate study interpretation. Therefore, developing a reliable large animal model for inner ear drug delivery is crucial. The inner and middle ear anatomy of domestic pigs closely resembles that of humans, making them promising candidates for studying inner ear pharmacokinetics. However, unlike humans, the anatomical orientation and tortuosity of the porcine external ear canal frustrates local drug delivery to the inner ear.

**Methods:** In this study, we developed a surgical technique to access the tympanic membrane of pigs. To assess hearing pre- and post-surgery, auditory brainstem responses to click and pure tones were measured. Additionally, we performed 3D segmentation of the porcine inner ear images and used this data to simulate the diffusion of dexamethasone within the inner ear through fluid simulation software (FluidSim).

**Results:** We have successfully delivered dexamethasone and dexamethasone sodium phosphate to the porcine inner ear via the intratympanic injection. The recorded auditory brainstem measurements revealed no adverse effects on hearing thresholds attributable to the surgery. We have also simulated the diffusion rates for dexamethasone and dexamethasone sodium phosphate into the porcine inner ear and confirmed the accuracy of the simulations using in-vivo data.

**Discussion:** We have developed and characterized a method for conducting pharmacokinetic studies of the inner ear using pigs. This animal model closely mirrors the size of the human cochlea and the thickness of its barriers. The diffusion time and drug concentrations we reported align closely with the limited data available from human studies. Therefore, we have demonstrated the potential of using pigs as a large animal model for studying inner ear pharmacokinetics.

## 1 Introduction

Hearing loss is a significant sensory impairment that affects millions of individuals worldwide. Notably, it is also linked to the development of dementia in older adults, and addressing hearing loss may offer a safe means to reduce the risk of dementia (Livingston et al., 2020; Fr et al., 2023). While hearing aids and cochlear implants provide valuable tools for restoring some degree of hearing, clinical outcomes are variable. Neither hearing aids nor implants fully address all aspects of life quality, such as enjoying music and hearing in noisy environments. Cochlear implants necessitate intricate surgical procedures and may cause loss of residual hearing, especially at low frequencies. The complexity and high cost of the implantation surgery limit widespread adoption. Consequently, there is a growing demand for drugs and therapies that can both prevent loss and restore hearing.

Despite extensive research efforts aimed at pioneering pharmaceutical therapies to prevent or treat hearing loss, existing treatments, at best, provide only partial hearing restoration. The repeated local administration of steroids in cases of idiopathic sudden hearing loss is one example of such a partially effective therapy (Chandrasekhar et al., 2019; Plontke et al., 2022). When developing treatments for inner ear conditions, the therapeutic outcome depends not only on drug targeting but also on the delivery mechanism and bioavailability to the targeted tissue. One of the challenges of drug delivery is a generally limited understanding of inner ear pharmacokinetics, making it difficult to distinguish between treatment ineffectiveness and inadequacy of drug delivery. Common issues regarding drug delivery efficiency to the inner ear emanate from a lack of understanding about the quantity of drug that traverses the tympanic membrane (TM), is cleared by the middle ear before reaching the round window membrane (RWM), transverses the RWM, and diffuses in the cochlear perilymph distally to reach the cochlear apex to affect the target cells.

Studying inner ear pharmacokinetics in humans presents challenges that are hindered by the inability to sample inner ear fluids without invasive surgery. A limited number of studies collected samples from the human inner ear before cochlear implant insertion (Bird et al., 2011; 2007). However, this sampling is not spatially correlated to the cochlear regions since the sampling process itself can cause notable disturbances in the spatial distribution of the drug within the inner ear. Small animals are frequently used for studies on inner ear pharmacokinetics. However, rodent models and even non-human primates are problematic as the anatomy and size of the inner ear and its protective barriers do not match those of humans (Felix, 2002; Dai et al., 2017; Moatti, et al., 2023b). The RWM is 5–6 times thinner in rodents versus humans; therefore, inferring transport characteristics of drugs between the two species is challenging. Furthermore, the drug distribution in the inner ear is massively affected by the dimensions of the cochlear spirals. The length of the cochlea is much smaller in rodents such as mice or guinea pigs (7 mm and 18.5 mm, respectively) than in humans (measured as 29 mm from scala tympani or 32–34 mm from the top of pillar or hair cells) (Gummer et al.,1981; Burda et al., 1988; Hatsushika et al., 1990; Thorne et al., 1999). Consequently, the size mismatch between the species creates a significant challenge for detailed evaluation of therapeutic bioavailability, i.e., if the drugs reach their target. Thus, there is a need for a more suitable large animal model for inner ear drug delivery optimization and therapeutic efficacy testing. The ideal animal model would feature similar inner ear anatomical size, developmental stages, and frequency ranges of hearing to humans.

Recently the porcine model has emerged as a promising animal model to study inner ear pharmacokinetics and to develop cochlear implants and devices to continuously deliver drugs to the middle ear (Yildiz et al., 2022; Yildiz et al., 2023). The porcine inner ear anatomy, barrier thickness, and auditory frequencies are very similar to those of humans. For example, the porcine RWM thickness (∼100 µm) is closer to humans (∼70 µm) than rodents (∼10 μm; Moatti et al., 2023a; Goycoolea, 2001; Goycoolea et al., 1987; Nordang et al., 2003; Tanaka and Motomura, 1981). The porcine basilar membrane length (measured from the top of hair cells) of 33.5 ± 5.0 mm (mean ± SD) and its 3.5 turns is also very similar to those of humans, ∼33.5 mm and 2.75 turns, and appears to be closer than other large animal models such as sheep (Kaufmann et al., 2020; Moatti et al., 2020). We have also studied the cochlear developmental stages (∼115 gestation days) in pigs and revealed their similarity to humans where the hearing organ is fully mature at birth, unlike mice (Moatti et al., 2022). Additionally, the frequency ranges of hearing (32–45,000 Hz) are close to humans (Lovell and Harper, 2007). The successful commercial cochlear implant insertion in porcine cochlea confirms the suitability of this large animal model for otologic studies (Yildiz et al., 2022). However, the narrow and angled conformation of the porcine ear canal makes it challenging to locally deliver drugs to the inner ear.

Here we address the challenges of working with pigs as an animal model by 1) developing a surgical approach for intratympanic injection in domestic pigs aged 4–8 weeks, bypassing the porcine ear canal; 2) adapting the bone conduction auditory brainstem responses (ABR) protocol to enable auditory assessment before and after surgery; 3) providing a detailed protocol for perilymph sampling at the study endpoint; 4) modifying a fluid simulation software (FluidSim) based on the unique anatomical measurements of pigs, assuming that perilymph flow operates in the order of nL/s. We employ this refined methodology to measure the concentrations of dexamethasone and dexamethasone sodium phosphate (DSP) in porcine perilymph and make comparisons with human data as well as with fluid simulation results. In essence, our proposed methodology can significantly enhance understanding of inner ear pharmacokinetics, with the expectation that leveraging the porcine model will facilitate the translation of novel testaments for human applications.

## 2 Materials and methods

### 2.1 Animals and anesthesia

The Institutional Animal Care and Use Committee at North Carolina State University (NCSU IACUC #22-118) approved this study. All methods were conducted according to the national guidelines under which the institution operates, and NIH Guidelines for the Care and Use of Laboratory Animals (8th edition). Ten, 2–4 weeks-old female Yorkshire piglets were included in this study. The mean animal body weight (BW) was 6.1 ± 1.52 kg (mean ± SD) at the time of surgery. Piglets had food withheld for 1–2 h prior to sedation and induction of anesthesia. For initial sedation, (TKX, 1.6–3.9 mg/kg body weight [BW] intramuscularly [i.m.]) a mix of tiletamine (50 mg/mL), zolazepam (50 mg/mL), ketamine (50 mg/mL), and xylazine (50 mg/mL) was injected behind the right ear. After a waiting period of 5–10 min, the animal was brought into an anesthesia preparation room and preoxygenated (10 L/min) for at least 5 min via a tight-fitting facemask with 100% oxygen at a flow rate of 10 L/min. Isoflurane (1%–5%, vaporized in 100% O_2_) was initiated if needed to maintain sedation. A venous catheter was inserted into an auricular vein and the piglet was intubated. The piglet was then transported to the operating room, where anesthesia was maintained on isoflurane (1%–5% vaporized in 100% O_2_). A constant body temperature between 38.3°C and 39.5°C was obtained using T/Pump water-circulated heat pads (Stryker, Minnesota, United States) and a bair hugger TM (3 M, Minnesota, United States). Intravenous balanced polyionic fluids (2.2 mL/kg/hr BW) were administered during anesthesia. Intraoperative pain medication consisted of 0.02 mg/kg BW buprenorphine. Locoregional anesthesia was provided using a mix of bupivacaine (1.25–2.5 mg/kg BW), epinephrine (0.005 mg/kg BW), and lidocaine (up to 4 mg/kg BW) in a subcutaneous line block at the intended surgery site.

### 2.2 Euthanasia in pigs

Following injection, general anesthesia was maintained for an additional 0.5 h to allow the perfusate to diffuse through the RWM prior to humane euthanasia with pentobarbital sodium (100 mg/kg BW intravenous [i.v.] or direct cardiac injection in anesthetized animals) and sample collection.

### 2.3 ABR

The stimulation and recording were controlled by an Intelligent Hearing Systems Duet with Smart EP (Miami, FL). Sound delivery was through an Etymotic speaker (ER-3b) connected by a sound tube to an in-ear insert. Bone conduction was through the B-81 Bone Vibrator. Needle electrodes were placed on the mastoid bone ventral to the stimulated ear (inverting) the vertex (non-inverting) and the forehead (ground). For bone conduction, the contralateral ear was masked with 80 dB nHl broad-band noise. Stimuli were primarily clicks although tone pips were also tested in some cases. Clicks were a 100 µs monophasic pulse and tones were 2 ms long with 1s rise/fall times. All stimuli were presented in alternating condensation and rarefaction phases, with up to 1,000 repetitions to each phase (fewer repetitions were used if the response was large). Levels for the transducers were calibrated by the manufacturers and are expressed in dB nHl relative to human hearing thresholds. The gain was 100,000x and recording epochs were 1,024 points collected in 25 ms.

### 2.4 Surgical tools

#### 2.4.1 Equipment

• Edmond Optics scientific camera (BFS-U3-13Y3C-C USB 3.1 Blackfly S) mounted (via a C-mount) on the Global Scope floor stand AXIS microscope.
• Anspach drill.

#### 2.4.2 Instruments

• Sterile non-reinforced surgical gown
• Sterile surgical gloves
• Half sheet converter (102 cm × 145 cm)
• Sterile rectal sleeve (alternatively sterilize drill piece/tubing)
• Sterile 3M Coban NL (3M St. Paul, MN)
• Sterile light handle covers
• Monopolar cautery
• Bipolar cautery
• Small animal standard pack
  o #3 scalpel handle
  o Adson Brown tissue forceps
  o Rat tooth tissue forceps
  o Mayo scissors
  o Metzenbaum scissors
  o Operating scissors (sharp-blunt)
  o Kelley forceps (2)
  o Crile forceps (2)
  o Mosquito forceps (4)
  o Rochester carmalt forceps
  o Towel clamps (4)
  o Needle holder
  o Huck towels (4)
  o Solution bowl
  o 4 × 4 gauze (20)
• Small Weitlander retractors
• Rongeurs
• Frazier suction tip 3Fr
• Frazier suction tip 7Fr
• Rat tooth forceps with the super fine tip
• Brown Adson forceps with the super fine tip
• Drill bit burr tips
• 20 GA 1-inch BD Insyte IV catheter
• 22G 1-inch needle
• Sterile huck towels
• Umbilical tape ¼ inch
• Sterile 0.9% saline (500 mLs)

### 2.5 Drugs

The dexamethasone sodium phosphate (DSP) J64083.06 was purchased from ThermoFisher and dexamethasone (Dex) D4902 was purchased from Sigma. The Dex was dissolved in ethanol for a total of 10 mg/mL master batch and then diluted in sterilized deionized (DI) water 1:100 for a final concentration of 100 μg/mL. DSP solution was made at 4 mg/mL in sterilized DI water and filtered with a 40 µm syringe filter.

### 2.6 Perilymph collection

Perilymph was collected from the RWM immediately *postmortem*. Piglets were positioned in right lateral recumbency to facilitate collection from the left, operated side. The incision edges were retracted and bone rongeurs were used to gently disrupt the mastoid process of the temporal bone and expose the round window. The connection of the inner ear to the brain was kept intact to prevent leakage of cerebrospinal fluid. Blood or tissue fluid was cleared from the round window with gauze. A 5 μL microhematocrit capillary tube (Fisherbrand™, Norwood, MA) with a distal end core diameter ranging from 0.11 to 0.12 cm was introduced into the RWM. Perilymph filled the tube by capillary action with a time interval of approximately 60 s. This process was repeated until a total of five microhematocrit tubes were collected for a total of ∼20 µL (∼4 µL in each vial was collected) and stored on dry ice. The piglet was repositioned in the left lateral recumbency to access the right, unoperated, control side. A 5 cm retroauricular skin incision was made centered at the level of the lateral canthus of the right eye. The incision was extended through the fibromuscular layers to access the mastoid process of the temporal bone. Perilymph collection was repeated as described previously. The perilymph samples were sent on dry ice in the hematocrit tubes (frozen) overnight to an outside vendor (Carbon Dynamic Institute) for mass spectrometry analysis. The samples from five tubes were pooled together for analysis after Dex injections. After DSP injections, for sequential sampling the first sample was not pooled with the other four samples, and the data is represented separately.

### 2.7 Tissue clearing and 3D imaging

The BoneClear procedure (Wang et al., 2019) was tailored for the porcine cochlea due to the size and intricacy of porcine tissues as previously described (Moatti et al., 2020; Li et al., 2021; Moatti et al., 2023b). The custom-built light-sheet microscopy was used for 3D imaging.

### 2.8 IMARIS segmentation

#### 2.8.1 Segmentation of structures within the inner ear

In IMARIS 9.5.1 using the “surface” tool under the “3D view” tab, a new surface was selected. This created a new surface in the scene for the data set. “Skip automatic creation, edit manually” was selected to start tracing along the slices of the structures to segment. The structures to be segmented for measurements were manually traced. The “contour” tab under the “draw” tab was selected to choose the orientation that best depicted the structures to be segmented under “board”. This can be the XY, YZ, or XZ planes. Switching to “mode”, the best drawing mode was chosen, in this case, the “distance pen” worked best. The “slice position” slider was used to move along the slices to trace along the structure of interest to be segmented. Once satisfied with the contour lines along the different slices, the “create structure” button was selected, which created the segmented structure. To segment a new structure, “surfaces” were chosen to start segmenting the new structure of interest.

#### 2.8.2 Measuring points

To measure lengths in the cochlea of 3D structures. The “measurement points” tool under the “3D view” tab was chosen to allocate points manually along the spiral trajectory. For instance, the brightly stained inner hair cells lining the organ of Corti were followed (stained against MYO7a) ensuring a ∼500 µm distance between different points.

#### 2.8.3 Quantification and statistical analysis

Once all the structures were segmented, the volumes of the structures were calculated using IMARIS. The structure of interest was selected to calculate the volume. Then, the “Statistics” tab was used to calculate all the statistics attributed to the previously selected structures. “Volume” was chosen from the drop-down menu and the volumes of the individual segments were displayed.

#### 2.8.4 Calculating distance versus cross-sectional area

Once the cochlear regions were segmented, the binary mask file was imported from IMARIS to MATLAB. For organs within the spiral, e.g., scala tympani, cochlear endolymphatic space, etc., a 3D logarithmic spiral was fitted to each volume (Pietsch et al., 2017). Then, a MATLAB script was used to generate a 2D slice out of the 3D volume that is perpendicular to the fitted logarithmic spiral progression. From the slice, the script measured the cross-sectional area every 100 µm. The distances versus area were calculated manually for scala tympani, cochlear endolymphatic space, and scala vestibuli - in locations that are not contained within the fitted spiral. This was done by going into a slice and adding contour lines around the region of interest. Once a contour line was created, “create structure” was selected and a mini segment was created, which underwent analysis to calculate the cross-section area. This process was repeated for the varying cross-sections.

## 3 Results

### 3.1 Intratympanic survival surgery in pigs

**FIGURE 1:**
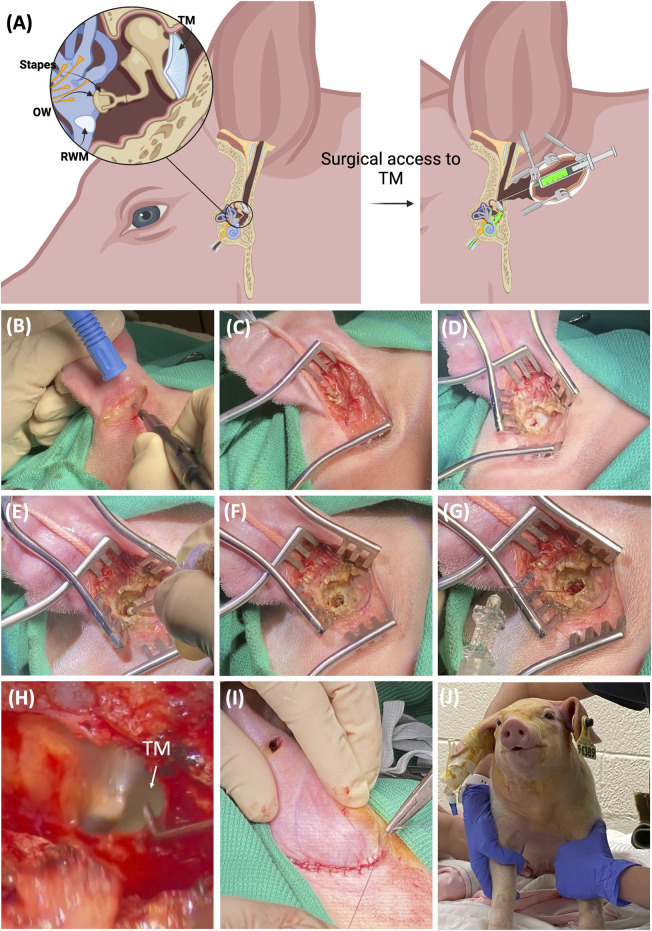
The intratympanic surgical procedure via transcanal approach in pigs. **(A)** The schematic of the porcine external, middle, and inner ear. With the removal of part of the external canal, the TM becomes accessible for the injection of the therapeutics into the middle ear cavity, which can then penetrate the inner ear via the RWM and the oval window (OW). **(B)** A 4 cm curvilinear incision was made 1–2 cm post auricularly using monopolar electrocautery in a 4-week-old piglet. **(C)** The underlying soft tissues were gently dissected and moved aside to reveal the cartilaginous outer ear. **(D)** Small Weitlaner retractors were used to open the incision and more tissue was removed to reveal the ear canal. **(E)** A 2 mm motorized fluted burr was used to facilitate the removal of the temporal bone. **(F)** The final view after the removal of the temporal bone reveals the TM. **(G)** A 6.3 cm (2.5 inch), 27 gauge needle was acquired and bent 3 mm from the tip to a 30-degree angle prior to being introduced through the TM to instill 1 mL of dexamethasone (0.1 mg/mL) or dexamethasone sodium phosphate (4 mg/mL) into the middle ear. **(H)** Higher resolution of TM via the surgical scope showing the injection site. **(I)** The incision was sutured closed for survival surgeries. **(J)** A piglet following recovery from general anesthesia.

As other groups have noted, there are anatomical differences between porcine and human external ear (Yildiz et al., 2022). The ear canal in pigs is tilted and narrow, which makes it almost impossible to access the TM directly down the external canal. Thus, a transcanal surgical approach was adopted and described to access the TM. The detailed surgical approach is demonstrated in Figure 1. Figure 1A shows the schematic of the porcine external, middle, and inner ear. With the transcancal approach and the removal of part of the external canal, the TM becomes accessible for the injection of the therapeutics into the middle ear cavity. The therapeutics can then penetrate the inner ear via the RWM and the oval window (OW). Supplementary Figure S1 shows the 3D scanned skull of a domestic pig depicting the inner ear in relation to the external ear canal detailing TM access. A step-by-step guide is provided below and in Supplementary Movies S1–S3:

1. The piglets were positioned in the right lateral recumbency, and a small surgical towel was used to tilt the head 45° sternal. All surgeries were performed on the left ear.
2. The surgical area, caudal to the lateral canthus of the left eye, rostral to the thoracic vertebra, from dorsal to ventral midline, was clipped using a #40 clipper blade.
3. The surgical area including the ear canal underwent careful cleaning to remove organic debris, followed by aseptic preparation using dilute betadine solution, and sterile draping of the body.
4. A 4 cm curvilinear incision was made 1–2 cm post auricularly using monopolar electrocautery (Figure 1B).
5. The underlying soft tissues were gently dissected and moved aside to reveal the cartilaginous outer ear (Figure 1C). Any hemorrhage was controlled using a combination of monopolar and bipolar cautery.
6. Small Weitlaner retractors were used to enhance exposure to the surgical site and dissection was continued to reveal the ear canal (Figure 1D).
7. The remainder of the surgery was performed under surgical microscopic guidance. The cartilaginous canal was transected at the transition between the cartilaginous and the bony wall in the ear canal using a number 15 scalpel blade (Figure 1D).
8. A 2 mm motorized fluted burr or rongeur was used to remove a portion of temporal bone sufficient to reveal the TM (Figures 1E, F). Light fluid irrigation with saline solution and suction using a 3 French fine Frazier suction tip was utilized to minimize thermal damage.
9. When the TM was visible, a 6.3 cm (2.5 inches), 27-gauge needle was acquired and bent 3 mm from the tip to a 30-degree angle prior to being introduced through the TM to instill 1 mL of Dex or DSP into the middle ear (Figures 1G, H). This volume exceeded the volume of the middle ear cavity resulting in some retrograde flow from the TM as evidenced in Supplementary Movie S3. Net surgical time was 38 ± 7 min (mean ± SD).
10. Following injection, general anesthesia was maintained for an additional 0.5 h to allow the perfusate to diffuse through the RWM prior to humane euthanasia and sample collection.
11. For survival surgeries, the incision was closed using 3-0 poliglecaprone 25 on a PS-2 reverse cutting needle (MONOCRYL™, Ethicon, Raritan, NJ) in a simple continuous suture pattern using 4-0 Vicryl sutures and covered with AluSpray aerosol bandage (Figures 1I, J).
12. Animals were fitted with an e-collar to minimize damage to the incision site. Each subject was closely monitored. The monitoring was performed up to 2 weeks postoperatively with routine incisional care and bandage changes. Throughout this period, the piglets remained in good health.

### 3.2 Auditory brainstem response (ABR) does not detect adverse hearing loss after intratympanic injection

We used ABR to evaluate the hearing of the animal and to make sure the surgical approach did not affect the animal’s ability to hear. Immediately after surgery, due to the temporary damage introduced to the TM and middle ear, an air-conduction ABR cannot be performed. Thus, a bone-conduction ABR was used as our outcome measure to assess damage to the inner ear postoperatively.

**FIGURE 2:**
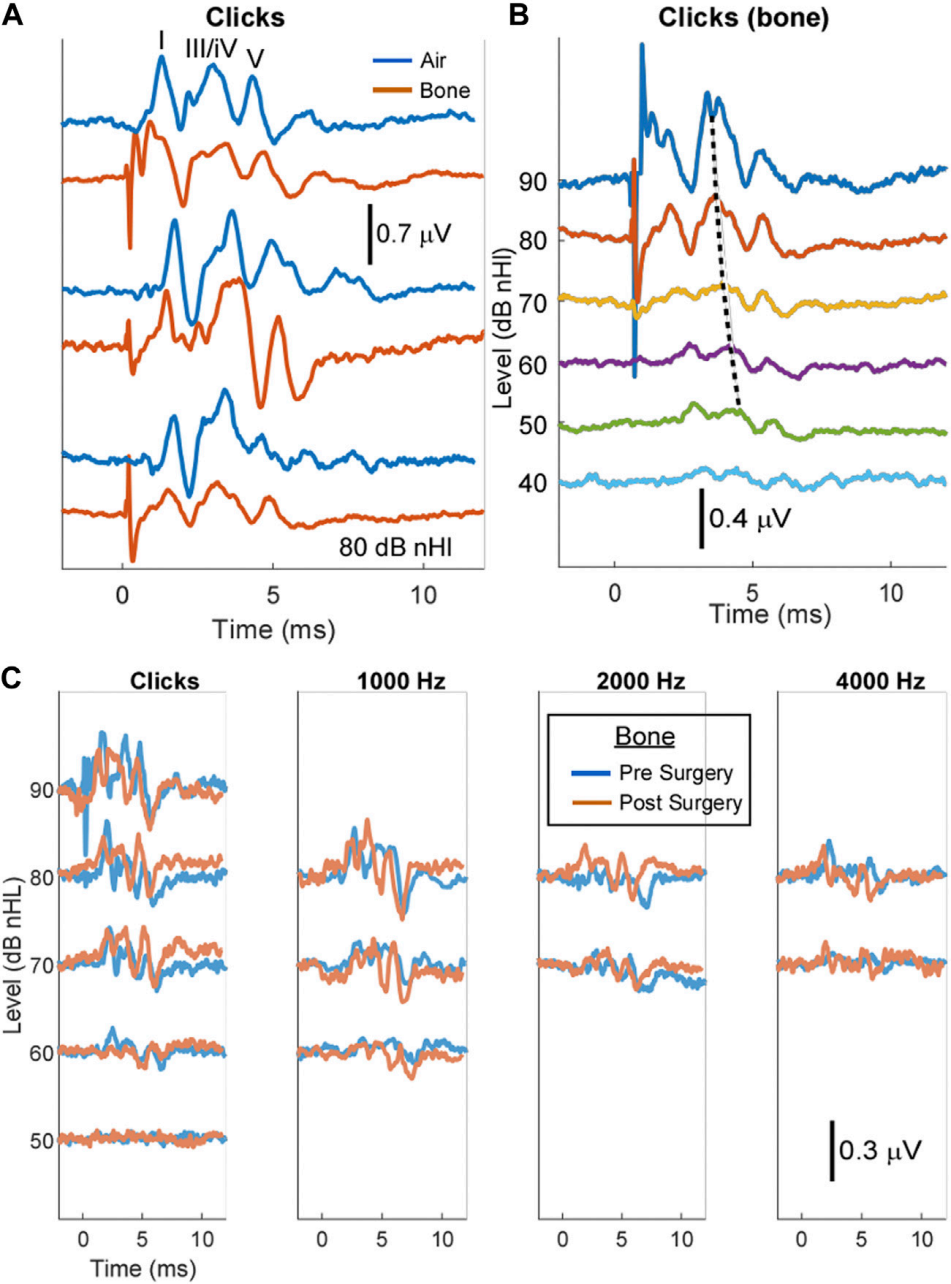
Auditory brain stem responses (ABR) before and after intratympanic injection show no adverse effect on hearing. **(A)** The air-versus bone-conduction ABR in a healthy pig shows all five peaks of the ABR. **(B)** The bone-conduction ABR threshold series in a healthy pig shows all five peaks of the ABR. The dotted black line shows the expected decrease in latency with increasing stimulus levels. **(C)** The click and pure tone bone-conductions ABRs at 1, 2, and 4 kHz before and after intratympanic injection showed no adverse effect on the threshold of hearing or general wave morphology.

First, we established the ABR measurements in the healthy pigs and compared air- and bone-conduction ABR. In Figure 2A, we show comparisons between air- and bone-conduction responses (blue and orange, respectively). The figure shows three different cases and in the top panel the three main peaks typically seen in the porcine ABR are shown (Hansen et al., 1992; Guo et al., 2010; Anderson et al., 2019). These three peaks are labeled as waves I, III/IV, and V with reference to human ABR waveforms. An example of a bone-conduction threshold series is shown in Figure 2B. The peaks show the expected decrease in latency with threshold increases (dotted line for wave III/IV). We found the air- and bone-conduction click-evoked ABR thresholds to be similar with mean values of 37 ± 4 dB (*n* = 3) and 38 ± 6.6 dB (*n* = 6) nHl, respectively. After surgery, there was no difference in the bone conduction thresholds (39 ± 10.0 dB nHL, *n* = 4). We observed click-evoked ABR bair-conducted latencies at 80 dB nHl to be slightly shorter than bone-conducted latencies. The latencies of wave I were 1.7 ± 0.5 (*n* = 8) compared to 1.9 ± 0.5 ms (*n* = 8), respectively, and latencies of wave III/IV were 3.3 ± 0.4 compared to 3.7 ± 0.4 ms, and latencies of wave V were 4.4 ± 0.4 compared to 4.8 ± 0.4 ms.

In Figure 2C, overlapped tracings are shown for clicks and tone stimuli with bone-conduction ABR before and after intratympanic injections. After surgery, the wave morphology and threshold to clicks were similar in both cases, and responses were present across the frequencies tested.

### 3.3 Dexamethasone and dexamethasone sodium phosphate exist in the perilymph of the inner ear after intratympanic delivery

Next, we evaluated the efficiency of our surgical approach with the delivery of the corticosteroids, dexamethasone (Dex) and dexamethasone sodium phosphate (DSP). The drugs were delivered in separate experiments to the middle ear through an intratympanic injection, i.e., either Dex or DSP was injected. The drug then passed from the middle ear through the RWM to the base of the inner ear. Although Dex passes through the RWM readily, the low solubility of Dex in solution is a significant issue, as its maximum solubility is low (0.1 mg/mL). To overcome the Dex solubility issue, DSP is often used; however, DSP has a lower permeability through the RWM. It is important to note that intratympanic injection of DSP is a common clinical procedure for the treatment of idiopathic sudden hearing loss (Chandrasekhar et al., 2019). In DSP the phosphate group is covalently bound to Dex, but through hydrolyses, DSP becomes enzymatically dephosphorylated to Dex (Derendorf and Hochhaus, 1995; Samtani et al., 2004; Samtani and Jusko, 2005). DSP is inactive until the phosphate group is cleaved by phosphatases. Therefore, when injecting DSP, both concentrations of Dex and DSP in the perilymph should be characterized.

**FIGURE 3:**
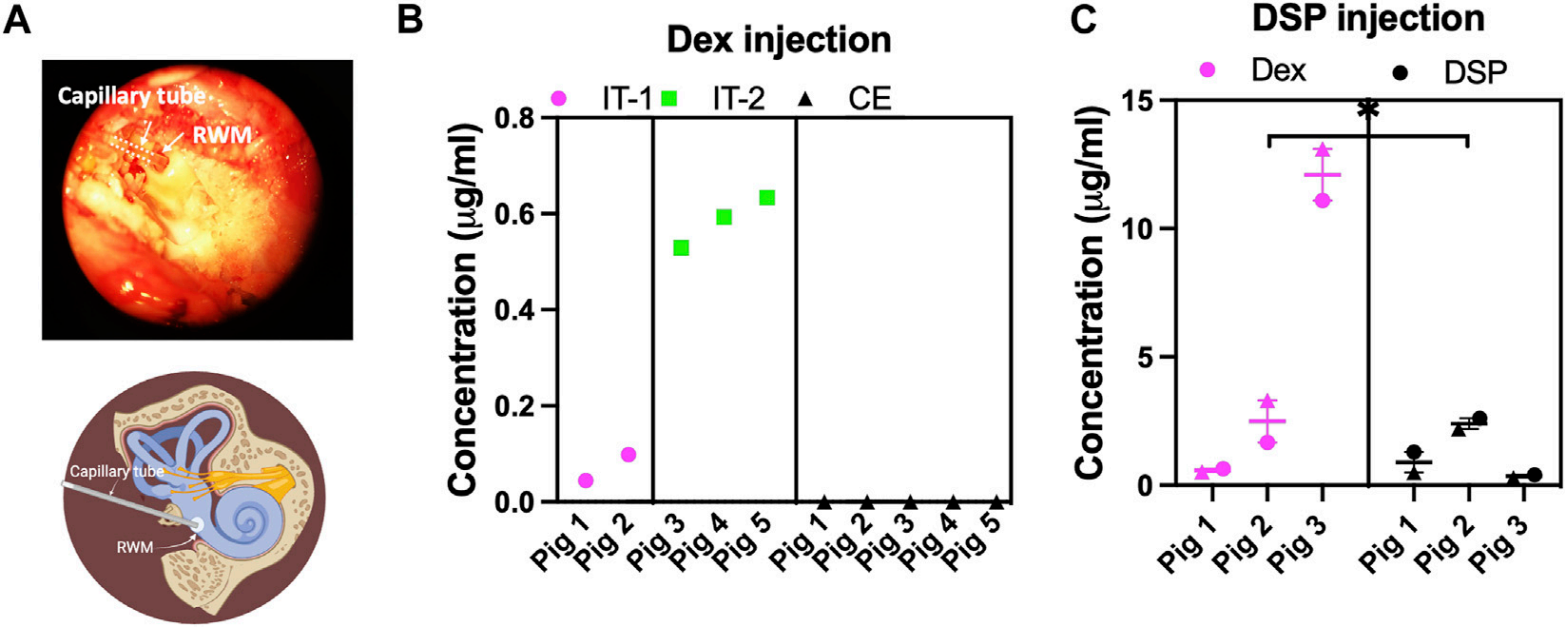
The intratympanic injection successfully delivers drugs to the porcine inner ear. **(A)** 20 µL of perilymph was collected from the RWM via a capillary tube 30 minutes post-injection. A schematic of the collection site is also provided. **(B)** The successful delivery of dexamethasone (Dex, 100 μg/mL) via intratympanic injection (*n* = 5) in two separate surgeries on two different days (IT-1 and IT-2) is shown. A significantly higher concentration of Dex in the injected ear (*p*-value = 0.0175) than the non-injected contralateral ear (CE). The Dex concentration was below the detection limit in the CE is observed. **(C)** The successful delivery of DSP (4 mg/mL) (measured as DEX after dephosphorylation and non-dephosphorylated DSP content) in the porcine inner ear via intratympanic injection (*n* = 3). For each piglet, two perilymph collections (a 5 µL sample showed by a circle symbol and a 15 µL sample showed by a triangle symbol) in consecutive order were performed and quantified using mass spectrometry, showing no difference between the collections. All data on graphs are shown as mean ± SEM. We used an unpaired t-test for comparison between groups, a *p*-value < 0.05 is considered significant. Note the difference in y-axis scales in **(B)** vs. **(C)**.

In non-survival procedures, piglets were euthanized 30 minutes after the injection, and perilymph (roughly 20 µL) was sampled from the RWM using microcapillary tubes (5 µL), as shown in Figure 3A (see also methods section). For clarity, a schematic of the perilymph collection from the RWM is also provided in Figure 3A. The concentrations of Dex and DSP (Dex injected at 100 μg/mL and DSP injected at 4 mg/mL) in the perilymph were measured using mass spectrometry. Figures 3B, C show the drug concentrations in the perilymph following treatment with Dex and DSP, respectively.

Figure 3B shows that Dex was present in the inner ear after intratympanic injection, but not in the perilymph of the contralateral ears (*p*-value: 0.0175, *n* = 5). Note, that in survival surgeries at one- and two-weeks post-surgery, no Dex was detected in the perilymph, which indicated a complete removal of Dex (*n* = 2). This was expected as Dex is cleared rapidly from the middle and inner ear of small rodents. The data presented in Figure 3B was obtained from two different surgery dates (IT-1 and IT-2). We found the mean ± SD for two surgeries performed at the same date to be 0.07 ± 0.04 (*n* = 2) while the mean for the other three surgeries in another date was 0.58 ± 0.05 μg/mL (*n* = 3).

After solely DSP injection, both DSP and dephosphorylated DSP (as Dex) content in the perilymph were measured. There was significantly more Dex content than DSP in the inner ear (*p*-value: 0.0256). To capture the gradient differences between the base and apex of the cochlea, perilymph from RWM was collected in two consecutive orders (sequential sampling from the base) that are shown by two separate data points per piglet in the graph. Most probably due to the sampling from the base, there was no difference in concentration of the drug after DSP injection between the 1st (the first 5 µL sample of perilymph from the base of the cochlea) and 2nd (a 15 µL sample) collection using an unpaired t-test (mean ± SD DSP content: 1.43 ± 1.10 versus 0.99 ± 1.04 μL/mL and mean ± SD Dex content: 4.47 ± 5.76 versus 5.64 ± 6.61 μL/mL, respectively).

### 3.4 3D segmentation of the porcine inner ear and incorporation of the pig model in FluidSim software

FluidSim software is used to simulate the diffusion of drugs within the perilymph of multiple animal models and various delivery methods including systemic and intratympanic application (Salt, 2002; Plontke et al., 2007; Lehner et al., 2023). FluidSim estimates the middle ear kinetics, round and oval window drug entry, drug elimination to the vasculature, and more. To incorporate the porcine model into FluidSim, cross-sectional data of various structures in the inner ear was required. The cross-sectional data needed to be taken along the longitudinal diffusion axis of the drug. To obtain this data, an intact porcine inner ear was tissue-cleared and 3D imaged using a custom-built light-sheet microscope as previously described (Moatti et al., 2023b).

**FIGURE 4:**
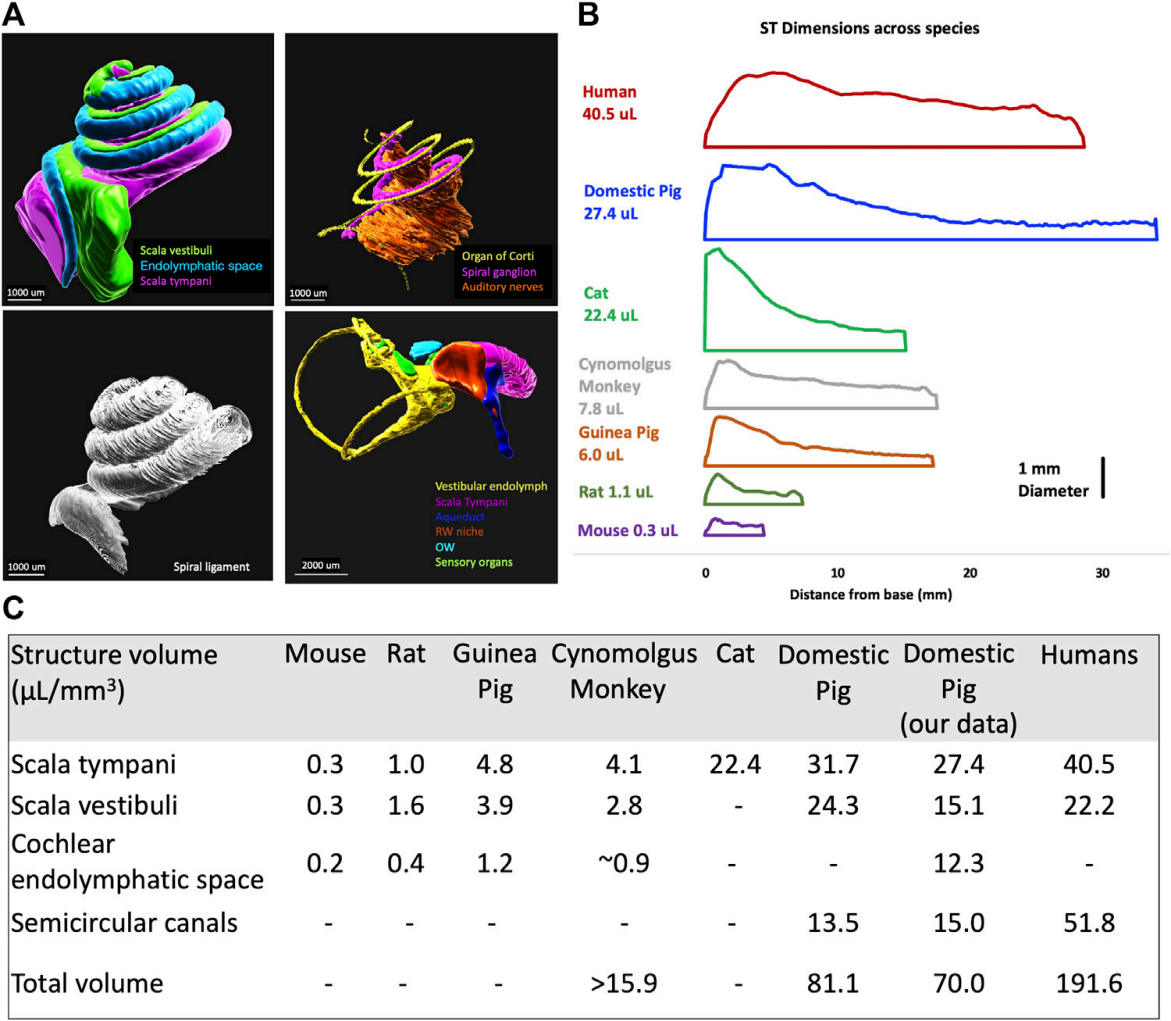
Volumetric segmentation of the porcine inner ear of a 4-weeks-old pig and newborn piglet. **(A)** 3D segmentation of inner ear organs; scala vestibuli, cochlear endolymphatic space, scala tympani, organ of Corti, spiral ganglion, auditory nerve, spiral ligament, vestibular endolymph, aqueduct, round window membrane (RWM), oval window (OW), and sensory organs of vestibular. **(B)** The scala tympani (ST) volume comparison between different species shows the similarity of pigs to humans. **(C)** The comparison of volumes of different inner ear structures between different species shows the similarity of the cochlea in pigs to humans. (Hatsushika et al., 1990; Thorne et al., 1999; Hiller et al., 2020; Manrique-Huarte et al., 2021; Yildiz et al., 2022).

IMARIS was used to segment in 3D different organs in the inner ear as shown in Figure 4A. From the 3D segmented structures, the cross-section data was derived using IMARIS and MATLAB (see *Methods* section) and incorporated into FluidSim code. Figure 4B shows a 2D representation of the scala tympani across species, the x-axis represents the length of the organ, and the y-axis represents the equivalent tube diameter, where the diameter (d) is calculated as $d=2\sqrt{\frac{Cross-Section\;Area}{\pi }}$ . The comparison of the other segmented volumes between pigs, humans, and other species is shown in Figure 4C. This data confirms the similarity of porcine cochlear volumes to humans. The vestibular volume; however, is much smaller in pigs than in humans.

**FIGURE 5:**
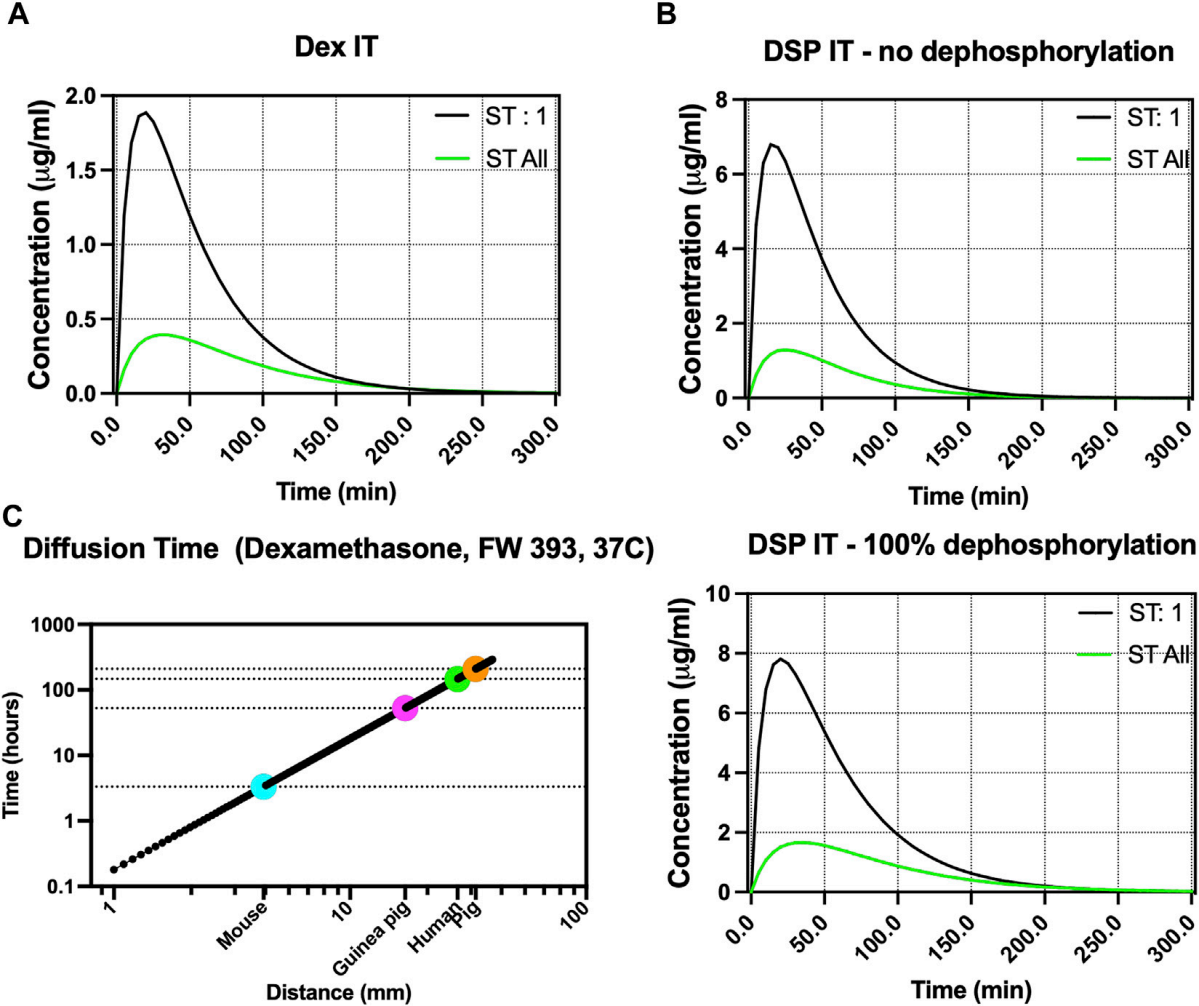
Fluid simulation of Dex and DSP in the porcine perilymph after intratympanic injection. **(A,B)** Simulation of Dex and DSP diffusion in the porcine scala tympani (ST) using our calculated cross-section area, respectively. Presumably, ST:1 refers to ST 1 mm from the base, and ST All refers to concentration summed across the entire ST. The RWM permeability values were measured using an *ex vivo* porcine RWM chamber. DSP is simulated for both conditions when DSP has no dephosphorylation and when DSP is 100% dephosphorylated in the inner ear. **(C)** Simulation of Dex diffusion from the base to apex across different species shows a total diffusion time of ∼8 and ∼6 days in pigs and humans, respectively.

We used the segmentation data and simulated the diffusion of Dex and DSP in the porcine inner ear. The RWM permeability measurements were obtained using an *ex vivo* porcine model (123 × 10^−9^ m/s for Dex and 12 × 10^−9^ m/s for DSP) and other parameters were set to the default FluidSim parameters (Moatti et al., 2023a). The Dex and DSP concentration as a function of time from injection and location along the scala tympani (ST) is shown in Figure 5A for a total of 300 min after intratympanic injection.

FluidSim predicted 30 min after intratympanic injection of Dex, the total amount of Dex in the whole scala tympani to be 0.39 μg/mL. At 1 mm away from the base, 30 min after delivery, FluidSim predicted that the Dex concentration would be 1.72 μg/mL.

FluidSim also predicted 30 min after intratympanic injection of DSP, the total amount of dephosphorylated DSP (if all hydrolyses) and DSP (if none hydrolyses) in the whole ST to be 1.65 and 1.27 μg/mL, respectively. At 1 mm away from the base, 30 min after delivery, FluidSim predicted that the Dex and DSP concentration would be 7.32 and 5.84 μg/mL, respectively.

Figure 5C shows the total diffusion time for Dex to reach the apex from the base of the cochlea in different animal models. The simulation of diffusion of Dex from the base to the apex of the cochlea shows a total diffusion time of ∼8 days and ∼6 days in pigs and humans, respectively.

Additionally, we measured the angle of the basal turn of the cochlea against the horizontal plane in a porcine fetal head. The fetal head was tissue cleared and imaged in 3D using a light sheet microscope (Supplementary Figure S2) we found the angle to be ∼34°. This angle in humans is measured at about 34° ± 3.6° as well (Tang et al., 2018). Given the similarity in the angle between humans and pigs, if there is an influence on fluid diffusion, the effect should be similar.

### 3.5 The cochlear aqueduct in pigs

The cochlear aqueduct, situated within the petrous temporal bone’s bony labyrinth, serves as a conduit for the perilymphatic duct. This duct facilitates the drainage of perilymph into the subarachnoid space of the posterior cranial fossa, merging with the cerebrospinal fluid (CSF).

We have conservatively measured the aqueduct dimensions from our 3D images. Tracing the aqueduct bone, i.e., without any soft tissue, yielded a diameter at the narrowest part of the aqueduct to be about 200–400 µm. We observed that the soft tissue occupies space within the aqueduct, as shown in Supplementary Figure S3, and therefore effectively decreasing the flow rate. As such 200–400 µm diameter is a conservative and overestimated measurement. The extent to which soft tissue is affected by the tissue-clearing process is unknown. This value of the bone diameter without consideration of the soft tissue is in line with the results reported in the literature (Yildiz et al., 2022). These values might not be substantially wider than those reported for humans 138 (±58) μm (Gopen et al., 1997).

## 4 Discussion

### 4.1 Porcine model for analyses of drug delivery translatable to humans

The development of a translatable large animal model for assessing intratympanic delivery could present an opportunity to improve the drug delivery of new and current therapeutics. The dimensions of the middle and inner ears, along with their associated barriers, significantly impact intratympanic delivery. While non-human primates have been used as the large animal model, from the ethical and financial perspective, their use in preclinical studies is limited and even banned in some places. The lower number of animals tested also results in lower statistical validity. On the other hand, the resemblance between the human and porcine middle and inner ears renders the pig an optimal large animal model for investigating intratympanic delivery. Nevertheless, the angled, narrow external auditory canal in pigs poses a challenge by obstructing direct access to the TM, thereby hindering the adaptation of simple intratympanic delivery in porcine models. To overcome this hurdle, we have developed and experimentally validated a surgical procedure for the safe intratympanic delivery of substances to the porcine inner ear (Figure 1).

This innovative approach offers the opportunity to evaluate a diverse range of promising therapeutics in a preclinical setting. For instance, in cancer patients, cisplatin therapy often results in hearing loss. To mitigate this debilitating side effect, systemic delivery of sodium thiosulphate was proposed. However, clinicians have commented on the potential for sodium thiosulphate to affect the survival rate when delivered systemically among patients with disseminated disease (Freyer et al., 2018; Viglietta et al., 2020; Witty and Cox, 2022). Local delivery of sodium thiosulphate could confine the drug predominantly to the inner ear, minimize any attenuation of cisplatin’s effects as needed for clinical applications in patients, and reduce the cost of treatment. The optimization of drug dosage, administration timing, and formulation could be facilitated in a predictable porcine model to reduce the cost of clinical trials. From an ethical standpoint, the potential expansion of data gathered from preclinical studies in pigs can enhance the robustness of results, aiding in the assessment of the feasibility and value of initiating human clinical trials.

The notion that local delivery achieves higher drug concentration in the perilymph and lower concentration in the plasma is supported by a 2007 study in humans (Bird et al., 2007). Not only did intratympanic delivery of Dex in humans produce an 88× higher concentration in the inner ear than systemic delivery, but also decreased the Dex concentration in plasma by 40×.

We’ve created and experimentally confirmed a surgical method for safely delivering therapeutics to the inner ear of pigs through intratympanic administration. This addresses the challenge presented by their angled and narrow external auditory canal.

### 4.2 Stable hearing threshold pre-and post-surgery

ABR measurements (Figure 2) were used as a tool to identify the safety of our procedure. Pure tone ABR of domestic pigs showed similar thresholds of 38 and 37 dB for air- (*n* = 3) and bone-conduction (*n* = 6) ABR, respectively. The bone-conduction ABR has been previously performed on minipigs (tone-burst thresholds were reported) but there is no report in standard pigs to the best of our knowledge (Yildiz et al., 2022). The observed differences between the air- and bone-conduction ABR can be attributed to the inherent differences between these two techniques. The bone-conduction ABR has been reported to be longer than the air-conduction ABR in humans (Sohmer and Freeman, 2001). Overall, the waves evoked by bone-conduction ABR in standard pigs were clear, stable, reproducible, and well differentiated. In other studies, air-conduction ABR thresholds were between 40- and 50-dB sound pressure levels (SPL) for Rongchang pigs and unknown breed pigs, not reported in the paper (Hansen et al., 1992; Guo et al., 2010; Anderson et al., 2019). Here, we observed a slightly lower threshed of 38 dB SPL (*n* = 3). The observed differences could be due to breed differences between piglets. Also, when comparing our ABR results to those reported for humans, wave V of the ABR in young piglets is about 8 ms, compared to 8–9 ms in children and 5 ms in older adults (Sharma et al.,2016).

Interestingly, in three cases, after the surgery, no ABR shift or change in the ABR morphology was observed in piglets confirming their intact hearing. In only one case we did record a 20 dB shift in the ABR threshold of hearing. We can attribute this case either to our surgical procedure or a change in electrode position; nevertheless, it emphasizes the importance of performing ABR before and after surgical procedures. For the next experiments, we plan to keep the electrode positions intact before and after surgery to minimize that source of variability. If the ABR shift is observed consistently in patients where the drill is used to remove the bone, the use of rongeur is suggested instead.

### 4.3 The efficacy of the surgical procedure

The mass spectrometry data (Figure 3) from the collected perilymph confirms the successful delivery of the drugs (Dex and DSP) to the inner ear. The low concentration of the Dex in the inner ear after Dex injection (0.38 ± 0.28 μg/mL, mean ± SD) is limited by the removal processes in the inner and middle ear including through the aqueduct and vasculature. The initial Dex concentration of 100 μg/mL is also limited due to the low solubility of Dex, which prevents the injection of higher drug concentrations. Significantly different concentrations of Dex inside the inner ear were recorded during the two surgeries, *n* = 5 (Figure 3B, 0.07 ± 0.04 versus 0.58 ± 0.05 μg/mL, mean ± SD). The observed variation could be due to the natural variability in the animals, contamination of Dex from the middle ear during perilymph collection, or surgical procedure variation such as head position. Any delay in sample collection after perilymph removal could result in lower drug concentration. This variation could be also related to the presence of a false round window membrane which could hinder the substance passage and therefore affect the total concentration of the observed drug in the perilymph. There is a report on the presence of fibrous plug and false round window membrane of 11% and 21%, respectively, in human patients causing the same problem clinically (Juan and Linthicum, 2010). A large range of Dex concentrations (0.03–95.2 μg/mL) is also reported after intratympanic injection of DSP in humans (Bird et al., 2007).

Thirty minutes after the DSP injection, we observed higher Dex (dephosphorylated) concentration than DSP inside the inner ear. It was previously reported that after intratympanic DSP (0.4–1.8 mL of a 4 mg/mL solution, *n* = 13) injection in humans, the concentration of Dex in the perilymph was 1.4 μg/mL (range, 0.1–16.3) compared to 7.5 μg/mL for DSP (range, 0.03–95.2) (Bird et al., 2007). In porcine inner ear after intratympanic injection of DSP (1 mL of 4 mg/mL, *n* = 3), we found the Dex concentration in perilymph to be 5.06 μg/mL (range, 0.52–13.10) and the DSP concentration to be 1.21 μg/mL (range, 0.25–2.60). We noticed a higher ratio of Dex to DSP in domestic pigs than in humans, which could be due to the higher dephosphorylation rate in pigs, or lack of stabilization of DSP after perilymph collection in our technique (not stored in the presence of phosphatase enzyme inhibitor such as ethylenediaminetetraacetic acid--EDTA). To clarify this issue, in the future, we plan to study the DSP to Dex conversion rate in the perilymph collected from the pigs using HPLC or mass spectrometry measurements. Overall, the mean total concentration of the Dex + DSP in the perilymph is very similar in both pigs and humans (6.3 versus 8.9 μg/mL) after equivalent intratympanic injections of DSP.

### 4.4 Simulation of drug diffusion in the inner ear

The porcine volumetric data of different cochlear structures (Figure 4) shows the likeness to humans. Specifically, the similarity between the size of scala tympani in humans and pigs would seem to make the diffusion predictions from base to apex in pigs more relevant than other animal models, including non-human primates. The volumes of the porcine inner ear that we calculated using 3D imaging were very similar to the data collected from micro-CT scans of domestic pigs (Yildiz et al., 2022). The predicted time of diffusion from the cochlear base to the apex was calculated to be 6 days for humans and 8 days for pigs. The vestibular volumes, however, are smaller in pigs confirming similarity between pigs and humans.

Using the volumetric data and derived cross-sections, FluidSim was adapted to the porcine model (Figure 5). Our porcine volumetric data is available at FluidSim4. Please note that other than the cross sections of cochlear organs, many experimentally determined parameters, such as RMW permeability and rate of elimination from the perilymph, can be adjusted in FluidSim to positively impact the simulation results. Therefore, as more experimental data becomes available for pigs, the FluidSim model can be increasingly refined for greater fidelity. For example, here we used the original setup in the FluidSim and changed the permeability of the RWM which seems to have the highest impact on the drug content in the perilymph. However, for fine-tuning, other factors such as drug elimination from the middle ear, elimination from scalas, CSF entry flow rate, oscillation, blood elimination, and more can be adjusted as well. Given our previous *ex vivo* permeability measurements for Dex and DSP and using default elimination rates from the middle and inner ear, FluidSim predicted the total amount of Dex, dephosphorylated DSP (if all hydrolyses), and DSP (if none hydrolyses) in the whole scala tympani to be 0.39, 1.65, and 1.27 μg/mL, respectively. Additionally, Dex, dephosphorylated DSP (if all hydrolyses), and DSP (if none hydrolyses) concentrations at 1 mm from the base are 1.7, 7.32, and 5.84 μg/mL, respectively. The values we experimentally found in the 20 µL of perilymph for Dex and DSP (total of DSP and dephosphorylated DSP) were 0.38 ± 0.25 and 6.27 ± 5.62 μg/mL (mean ± SD), respectively. Overall, the simulated values are close to the experimental values, and future work will optimize FluidSim for specific drugs. In general, accurate simulation will extremely be helpful in studying inner ear pharmacokinetics.

In an attempt to capture the gradient differences between base and apex, we collected perilymph samples sequentially starting from the base of the RWM. Although we expected to see drug concentration differences between the samples, in all animals injected with DSP, the drug concentration in samples from the base and further distally in the cochlea were equivalent (mean ± SD DSP content: 1.43 ± 1.10 versus 0.99 ± 1.04 μL/mL; Dex content: 4.47 ± 5.76 versus 5.64 ± 6.61 μL/mL, respectively). This indicates the sequential sampling from the RWM is not appropriate for observing the gradient differences in the biodistribution and apical sampling may be necessary to study gradient differences (Salt et al.,2006).

### 4.5 The CSF entry from the cochlear aqueduct

Our experimental results for the concentration of drugs in the perilymph suggest that there is no significant exchange of fluid between the CSF and perilymph while collecting perilymph. This conclusion is supported by the fact that we did not observe a significant difference between the sequential perilymph sampling (Figure 3C, first sample—5 μL, second sample—15 µL). If CSF would enter and dilute the perilymph after the first measurement, we would expect to see a drop in the drug concentration in the second sample. Since we did not observe this difference, we inferred the rate of the flow of the CSF to the perilymph in the event of the RWM puncture is not substantial. On the other hand, when collecting the perilymph from pigs, 5 µL can be extracted quite rapidly, ∼1 min, which is different from non-human primates. This relatively rapid perilymph extraction can be indicative of a higher CSF flow to the perilymph through the aqueduct in pigs than non-human primates. This point can be further studied in the future.

## Data Availability

The datasets presented in this study can be found in online repositories. The names of the repository/repositories and accession number(s) can be found below: https://alecsalt.com/index.php/simulations/fluidsim4.
