# Erratum: Surgical procedure of intratympanic injection and inner ear pharmacokinetics simulation in domestic pigs

**DOI:** 10.3389/fphar.2024.1384445

**Published:** 2024-03-04

**Authors:** Frontiers Production Office

**Affiliations:** Frontiers Media SA, Lausanne, Switzerland

**Keywords:** intratympanic, inner ear, fluid simulation, round window membrane, pigs, swine, pharmacokinetics

Due to a production error, there was a mistake in Figure 4 as published. The background was black rather than white. The corrected Figure 4 appears below.

**FIGURE 4:**
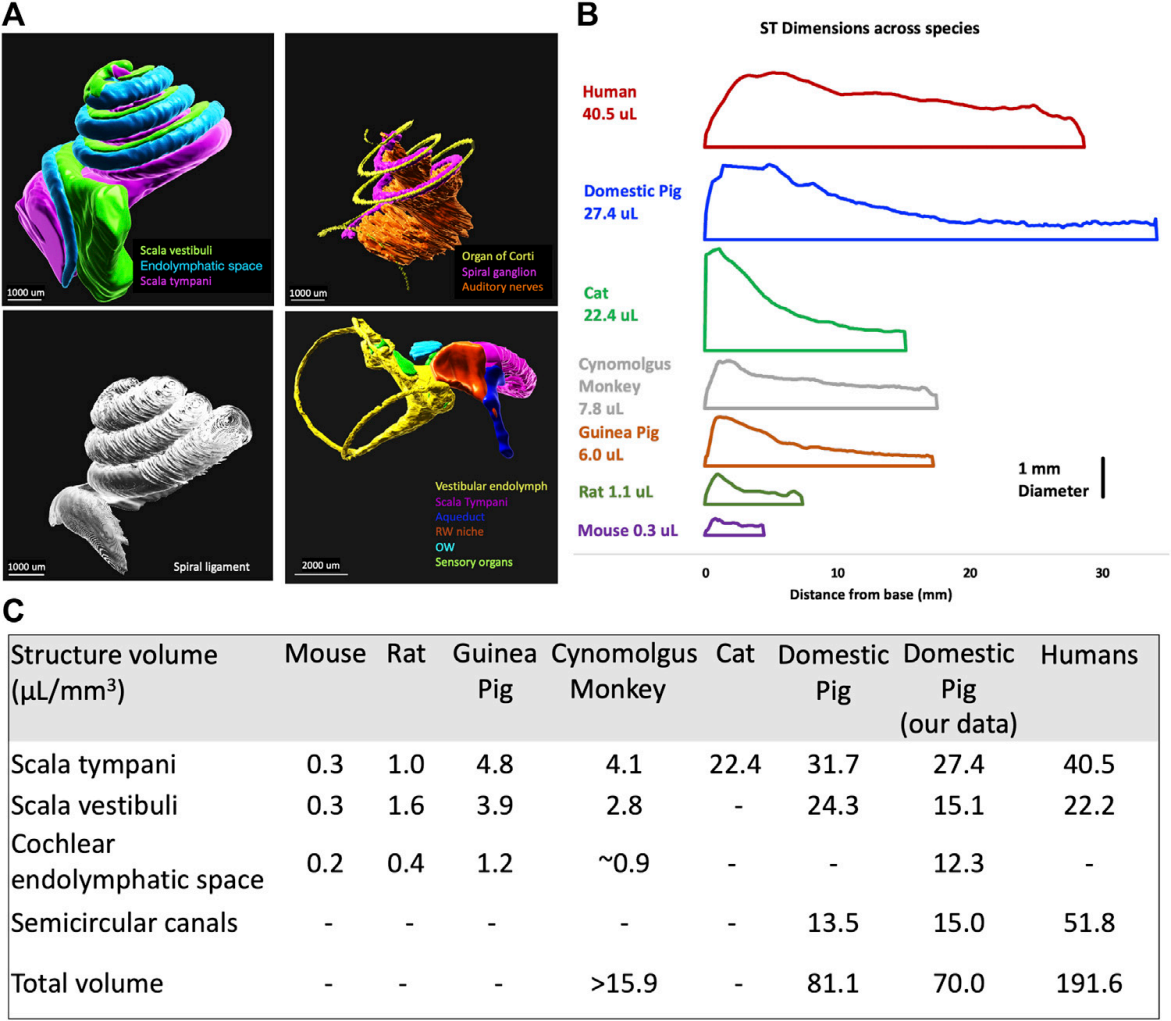
Volumetric segmentation of the porcine inner ear of a 4-weeks-old pig and newborn piglet. **(A)** 3D segmentation of inner ear organs; scala vestibuli, cochlear endolymphatic space, scala tympani, organ of Corti, spiral ganglion, auditory nerve, spiral ligament, vestibular endolymph, aqueduct, round window membrane (RWM), oval window (OW), and sensory organs of vestibular. **(B)** The scala tympani (ST) volume comparison between different species shows the similarity of pigs to humans. **(C)** The comparison of volumes of different inner ear structures between different species shows the similarity of the cochlea in pigs to humans. (Hatsushika et al., 1990; Thorne et al., 1999; Hiller et al., 2020; Manrique-Huarte et al., 2021; Yildiz et al., 2022).

The publisher apologizes for this mistake. The original version of this article has been updated.

