# Supplementary material for: Surgical procedure of intratympanic injection and inner ear pharmacokinetics simulation in domestic pigs
Source: Front Pharmacol. 2024 Jan 26;15:1348172. doi: 10.3389/fphar.2024.1348172 (PMC10853450; doi:10.3389/fphar.2024.1348172)
Supplement: Supplementary file 2 [file Table1.DOCX]

Supplementary Material

Surgical procedure of intratympanic injection and inner ear drug kinetic simulation in domestic pigs

Adele Moatti^1,2,^*, Shannon Connard^2,3^, Novietta De Britto^1,2^, William A Dunn^4^, Srishti Rastogi^1^, Mani Rai^1,2^, Lauren Schnabel^2,3^, Frances S Ligler^5^, Kendall A Hutson^4^, Douglas C Fitzpatrick^4^, Alec Salt^6^, Carlton J Zdanski^4^, Alon Greenbaum^1,^*

^1^Joint Department of Biomedical Engineering, University of North Carolina at Chapel Hill and North Carolina State University, Raleigh, NC 27606, USA

^2^Comparative Medicine Institute, North Carolina State University, Raleigh, NC

^3^Department of Clinical Sciences, North Carolina State University, Raleigh, NC 27606, USA

^4^Department of Otolaryngology- Head and Neck Surgery, University of North Carolina at Chapel Hill, Chapel Hill, NC 27599, USA

^5^Department of Biomedical Engineering, Texas A&M University, College Station, TX 77843, USA

^6^Tuner Scientific, Jacksonville, IL 62650, USA

*** Correspondence:**Adele Moatti, [amoatti@ncsu.edu](mailto:amoatti@ncsu.edu); Alon Greenbaum,[Greenbaum@ncsu.edu](mailto:Greenbaum@ncsu.edu)

**
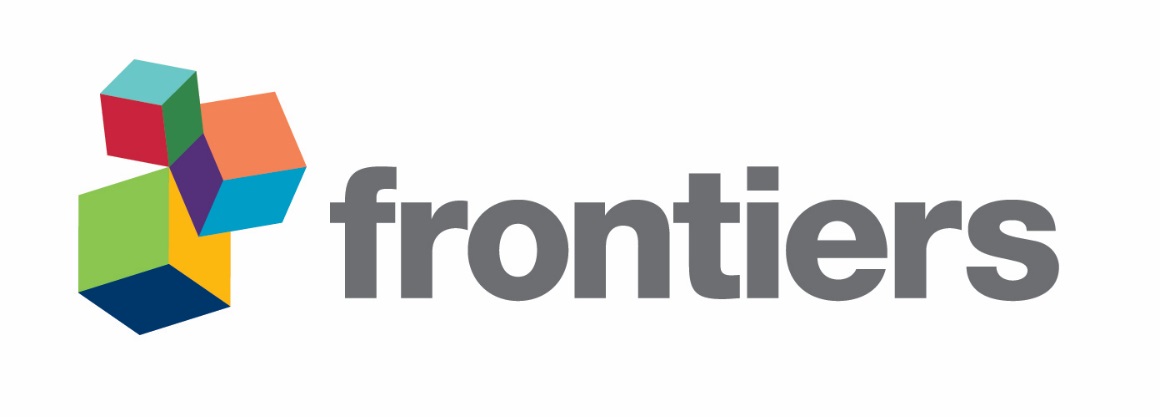
**

# Supplementary Figures and Tables

## Supplementary Figures

**
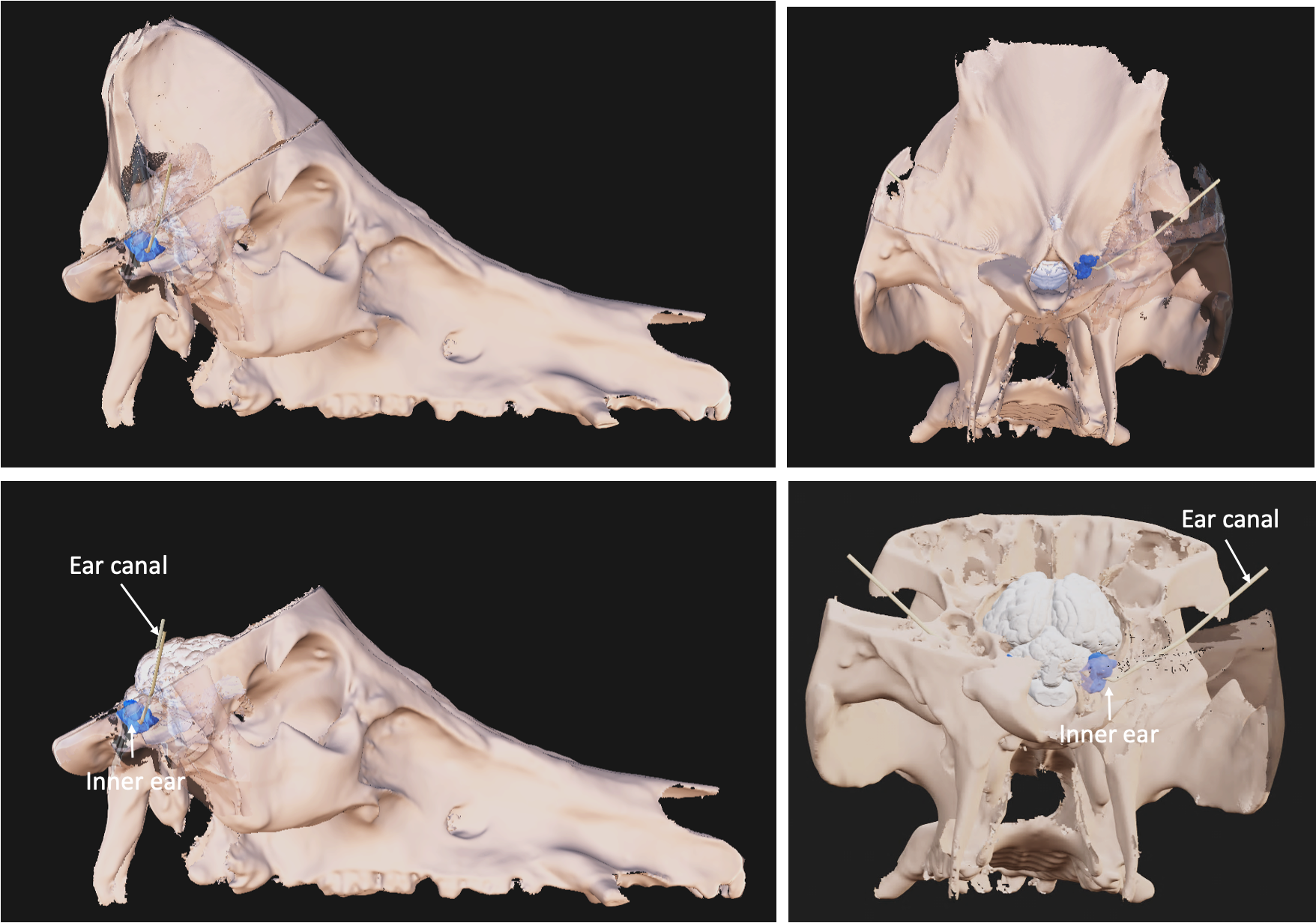
**

**Supplementary Figure 1.** The 3D-scanned porcine skull, inner ear, and brain show the relative position of the external ear canal and inner ear in the porcine skull from the lateral and posterior views. The bone on the right side in the proximity of the ear is made transparent for more clarity. The second row shows the ear canal and inner ear locations without the skull cap for more clarity.


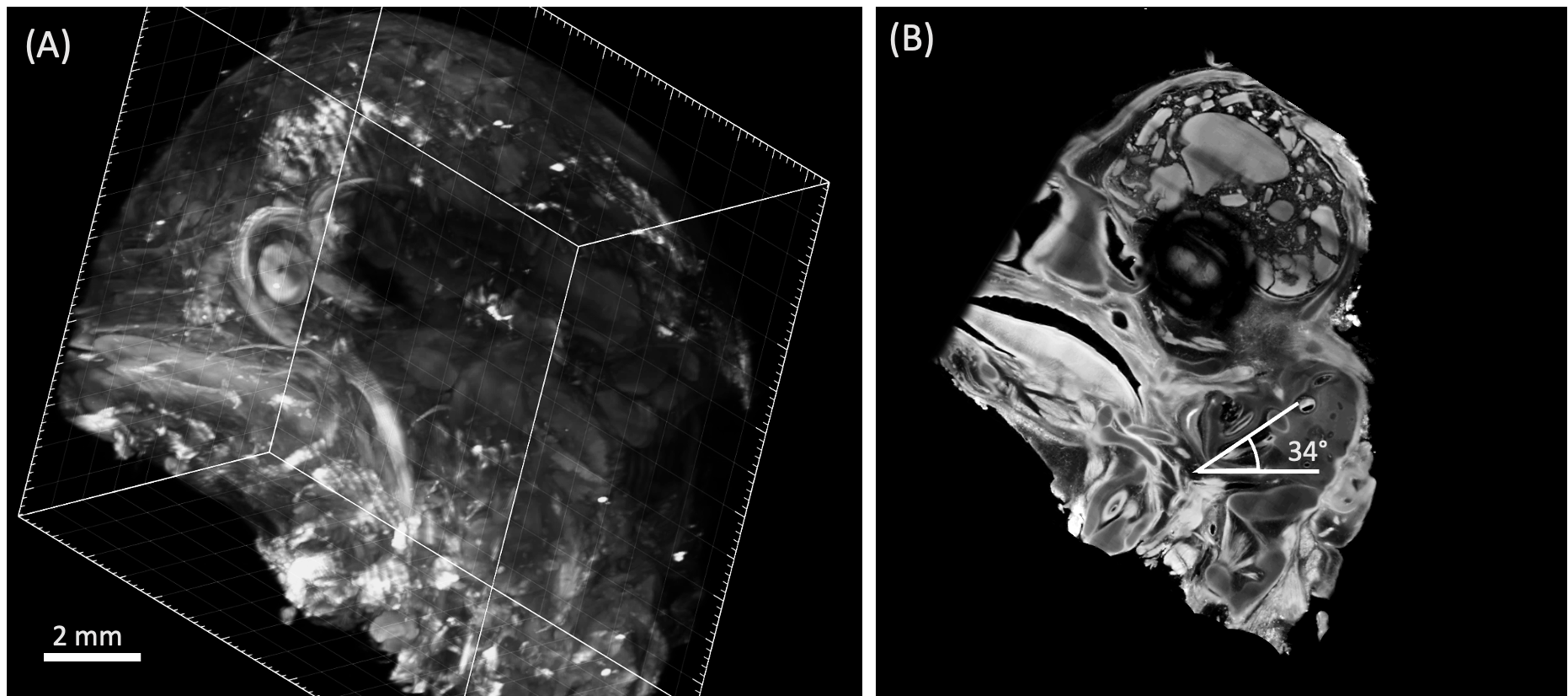


**Supplementary Figure 2**. (A) 3D image of an 80-day-old porcine fetus head captured with a light sheet microscope. The entire head was tissue-cleared using the BoneClear technique. (B) The cochlear position in the fetal head shows ~34 degrees between the basal turn of the cochlea section and the horizontal plane.


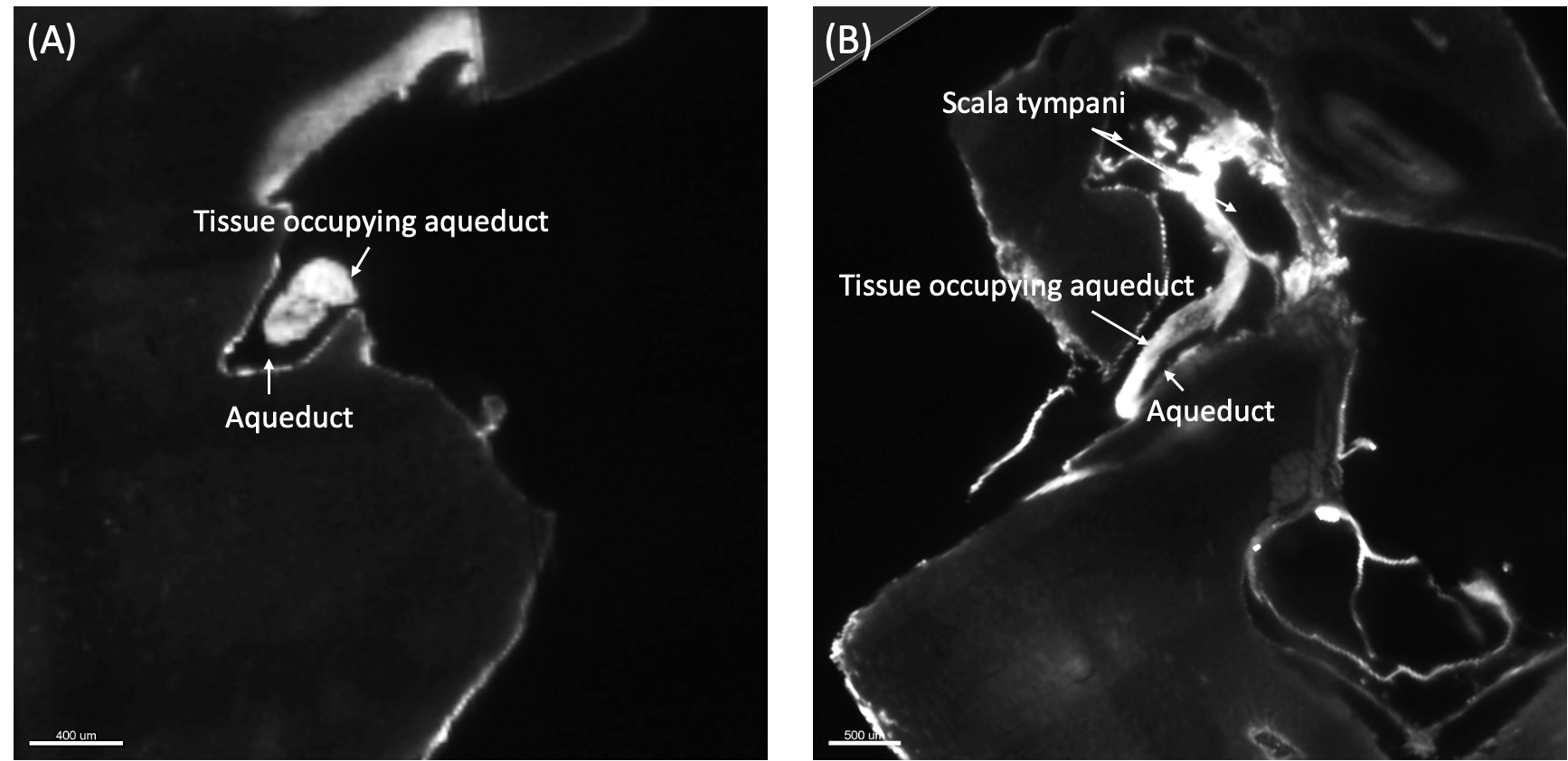


**Supplementary Figure 3.** The pig aqueduct is depicted in the following images. (A) A single z-plane (20 µm thickness) extracted from the 3D volume of a newborn pig. The presence of soft tissue within the aqueduct may significantly decrease its diameter. (B) Another z-plane (20 µm thickness) rotated 90 degrees in relation to (A). This view highlights the connection of the aqueduct to the Scala tympani, with soft tissue within the aqueduct potentially contributing to a reduction in its diameter.
